# A case of miliary tuberculosis following transurethral surgery and prostate biopsy after intravesical Bacillus Calmette–Guerin immunotherapy

**DOI:** 10.1002/iju5.12385

**Published:** 2021-10-03

**Authors:** Yoshiaki Kurokawa, Taketo Kawai, Jimpei Miyakawa, Naohiro Makise, Yoshiyuki Akiyama, Yuta Yamada, Yusuke Sato, Daisuke Yamada, Tetsuo Ushiku, Haruki Kume

**Affiliations:** ^1^ Departments of Urology Graduate School of Medicine The University of Tokyo Tokyo Japan; ^2^ Department of Pathology Graduate School of Medicine The University of Tokyo Tokyo Japan

**Keywords:** BCG immunotherapy, bladder cancer, miliary tuberculosis, *Mycobacterium bovis*, systemic infection

## Abstract

**Introduction:**

Intravesical Bacillus Calmette–Guerin immunotherapy is known to prevent recurrence of bladder cancer, but it can cause tuberculosis infections as an adverse event.

**Case presentation:**

A 75‐year‐old man visited our hospital due to hematuria. The patient was diagnosed with bladder cancer and underwent transurethral resection of the bladder tumor. Postoperatively, the patient received Bacillus Calmette–Guerin immunotherapy. One year later, we performed transurethral surgery and prostate biopsy because of cystoscopic findings showing nodulous lesions in the bladder and an elevated serum prostate‐specific antigen level. The patient presented with high fever and malaise since the surgery. After careful examination, the patient was diagnosed with miliary tuberculosis caused by *Mycobacterium bovis*. The pathology of the bladder and prostate revealed acid‐fast bacilli collection by Ziehl–Neelsen staining.

**Conclusion:**

The surgery exacerbated the local infection into a systemic infection. The risk of developing miliary tuberculosis should be considered at transurethral surgery or prostate biopsy in patients after intravesical Bacillus Calmette–Guerin immunotherapy.

**Abbreviations & Acronyms**
BCG: Bacillus Calmette–Guerin
CT: computed tomography
DNA: deoxyribonucleic acid
*M*. *bovis*
: *Mycobacterium bovis*
PCR: polymerase chain reaction
PSA: prostate‐specific antigen
TURBT: transurethral resection of the bladder tumor

**Keynote message**

This is the first report that after BCG immunotherapy, not only do local infections persist in the bladder and prostate, but transurethral surgery or prostate biopsy may also cause systemic dissemination.

## Introduction

Intravesical BCG immunotherapy is routinely used for the prevention of recurrence of non‐muscle invasive bladder cancer and for the treatment of carcinoma in situ, with strong evidence of efficacy.^1^ However, BCG is also known to be associated with a high incidence of adverse events, with dysuria and hematuria being the most common. In rare cases, systemic infections such as miliary tuberculosis, hepatitis, and arthritis may also occur and require attention.^2^

## Case presentation

A 75‐year‐old male presented to our department due to gross hematuria starting 9 months prior. Cystoscopy revealed a bladder tumor, and TURBT was performed. Histopathology revealed urothelial carcinoma, high‐grade, G2, pTa, ly0, and v0. Intravesical BCG induction immunotherapy was performed once a week for a total of 8 weeks. For each administration, 80 mg of BCG Tokyo‐172 strain was instilled into the bladder for 2 hours. During the courses, the patient had painful and frequent urination, and aseptic pyuria.

**Fig. 1:**
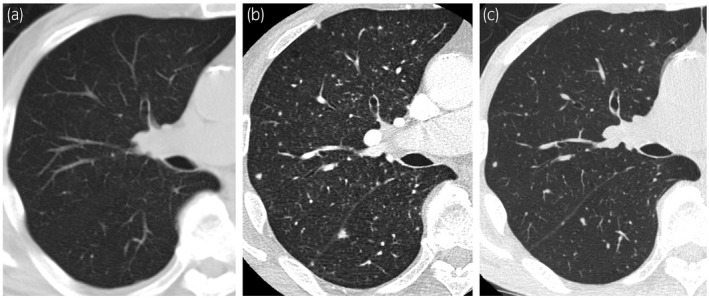
Chest CT findings. (a) No findings on the postoperative day 4. (b) Diffuse granular shadows were observed in bilateral lung fields, which was a typical finding of miliary tuberculosis. (c) Improvement of granular shadows after 4 months of treatment.

**Fig. 2:**
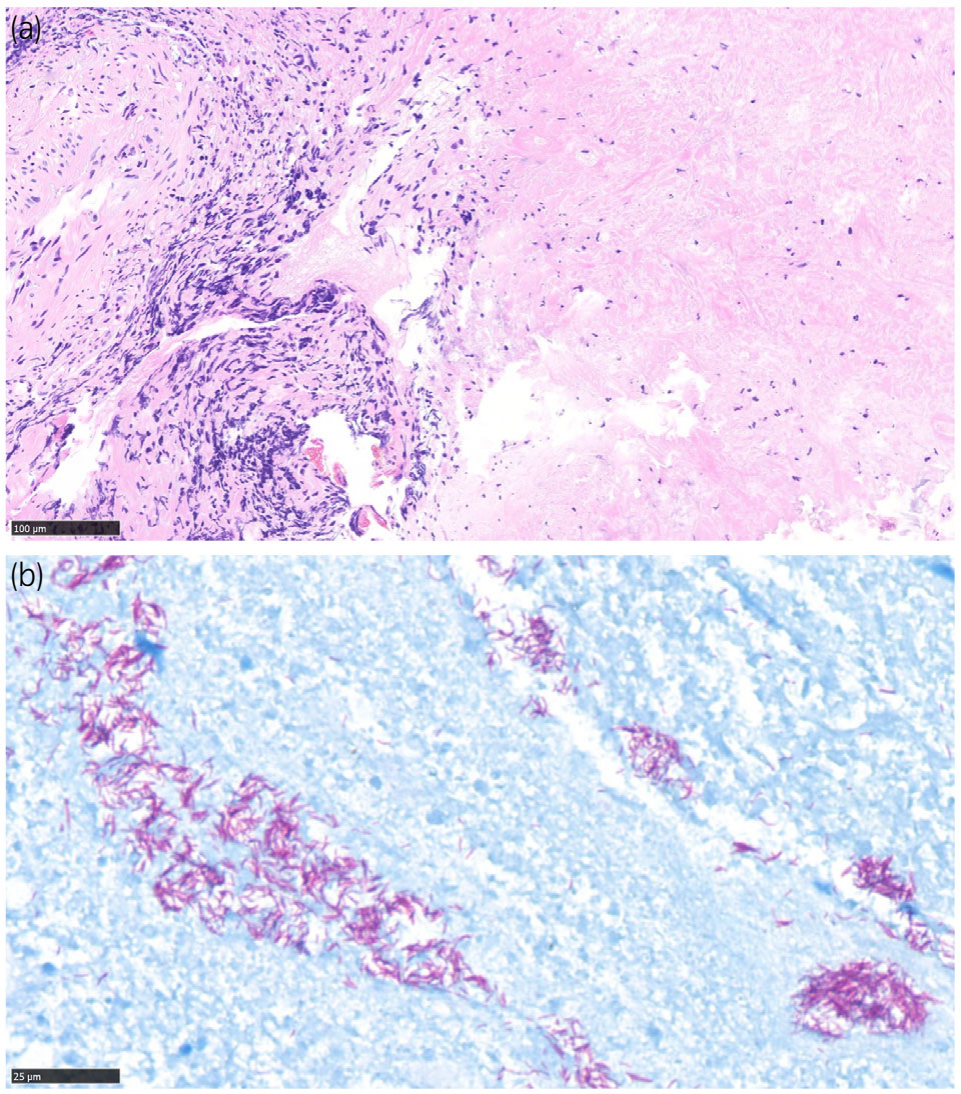
Pathological findings. (a) Hematoxylin and eosin staining reveals caseating granuloma in bladder biopsy specimen. The bar indicates 100 μm. (b) Ziehl–Neelsen staining revealed collection of acid‐fast bacillus. The bar indicates 25 μm.

Eleven months after the last BCG instillation, occasional urination pain and aseptic pyuria had persisted. Cystoscopy revealed nodulous lesions at the left ureteral orifice. CT showed left hydronephrosis, and serum PSA was elevated at 12.98 ng/mL. Considering the possibility of recurrence of bladder cancer and development of prostate cancer, transurethral biopsy of the bladder mucosa, retrograde urography, ureteroscopy, and transperineal prostate biopsy were performed. Immediately after surgery, the patient developed high fever and we started antibiotic therapy; however, the patient's temperature did not decrease. A CT scan was performed on the postoperative day 4, but the cause of the fever was unknown (Fig. 1a). Blood tests showed elevated liver enzymes and pancytopenia. A CT scan on postoperative day 14 showed diffuse granular shadows in the lungs, suggesting miliary tuberculosis (Fig. 1b). Pathology showed no malignant findings in either the bladder or prostate. Bladder biopsied specimens showed caseating granuloma (Fig. 2a), and Ziehl–Neelsen staining revealed collection of acid‐fast bacillus (Fig. 2b). The same findings were seen in the prostate.

On postoperative day 21, a urine PCR test revealed mycobacterium tuberculosis complex, which led to the diagnosis of miliary tuberculosis. We started treatment with isoniazid, rifampicin, ethambutol, and prednisolone. After the start of treatment, fever was resolved and liver enzymes improved. On postoperative day 54, *M. bovis* was identified by urine culture, and it was found that the infection was caused by BCG immunotherapy performed 1 year before the surgery. After 3 months of antituberculosis treatment, a urine PCR test of mycobacterium tuberculosis complex was negative. In addition, after 4 months of treatment, a CT scan showed improvement in diffuse granular shadows in the lung fields (Fig. 1c).

## Discussion

There have been several reports of infections associated with BCG immunotherapy. Asin *et al*. reported that 4.3% of patients developed systemic infections (miliary tuberculosis, hepatitis, nephritis, lymphocytic meningitis, arthritis, or osteomyelitis) due to BCG immunotherapy. In addition, 48% of patients with BCG infection showed the presence of M. bovis by either acid‐fast staining, culture, or PCR, and 86.3% showed granulomas on pathology.^3^

The pathway leading to systemic infection is thought to be hematogenous dissemination from the renal pelvis to the whole body, and Siatelis *et al*. reported that *M. bovis* BCG DNA was found in blood in 8.4% of patients 24 hours after BCG instillation.^4^ Conversely, for patients with miliary shadows on CT and granulomas on pathology but negative microbiologic diagnosis, the involvement of a type 4 hypersensitivity rather than bloodstream infection has been considered, and there have been reports of improvement with follow‐up alone.^5^, ^6^ According to Gonzalez *et al*., there are two types of BCG infections depending on the onset of symptoms: early‐onset BCG infections with systemic symptoms, which are more common within 3 months of instillation, and late‐onset BCG infections with local symptoms that appear more than 1 year later.^7^

BCG is known to persist after instillation, and Siatelis *et al*. reported that 1 of 10 patients had BCG PCR in urine 6 weeks after the last BCG instillation.^4^ Furthermore, Durek *et al*. reported that 4.2% of bladder biopsies taken 12 months after the last BCG instillation were positive for BCG PCR.^8^ Both reports suggest that BCG may persist in the bladder for a long time and cause late‐onset BCG infection, although the number of cases is small.

In the present case, approximately 1 year had passed since BCG immunotherapy. Preoperative urinalysis revealed aseptic pyuria. Pathology showed caseating granuloma in the bladder and prostate. Acid‐fast staining and urine culture showed *M. bovis*. Therefore, it was clear that the bladder and prostate were chronically infected before the surgery. The patient who had only local symptoms for 1 year presented with systemic symptoms immediately after surgery. In addition, miliary shadows on CT were not visible on postoperative day 4, but were clear on postoperative day 14. Because of these facts, it is presumed that the bloodstream infection caused by transurethral surgery or prostate biopsy resulted in symptoms such as fever, which led to miliary tuberculosis. There have been no similar reports, and it should be kept in mind that after BCG immunotherapy, not only do local infections persist in the bladder and prostate, but transurethral surgery or prostate biopsy may also cause systemic dissemination.

## Conflict of interest

The authors have no conflict of interest to declare.

## Approval of the research protocol by an Institutional Reviewer Board

This report was approved by the Ethics Committee at the University of Tokyo Hospital (Approval number: 3124). Informed consent to participate was obtained from the patient.

## Informed consent

Written informed consent was obtained from the patient for publication of the details of this medical case and any accompanying images.

## Registry and the Registration No. of the study/trial

Not Applicable.
